# Genomic analysis of host – Peste des petits ruminants vaccine viral transcriptome uncovers transcription factors modulating immune regulatory pathways

**DOI:** 10.1186/s13567-015-0153-8

**Published:** 2015-02-24

**Authors:** Siddappa Manjunath, Gandham Ravi Kumar, Bishnu Prasad Mishra, Bina Mishra, Aditya Prasad Sahoo, Chaitanya G Joshi, Ashok K Tiwari, Kaushal Kishore Rajak, Sarath Chandra Janga

**Affiliations:** Division of Veterinary Biotechnology, Indian Veterinary Research Institute, Izatnagar-243122, Bareilly, India; School of Informatics and Computing, Indiana University Purdue University, 719 Indiana Ave Ste 319, Walker Plaza Building, Indianapolis, Indiana 46202 USA; Department of Animal Biotechnology, College of Veterinary Science & Animal Husbandry, Anand Agricultural University, Anand, Gujarat 388001 India; Division of Virology, Indian Veterinary Research Institute (IVRI), Mukteswar Campus, Nainital (Uttaranchal), 263138 India; Center for Computational Biology and Bioinformatics, Indiana University School of Medicine, 5021 Health Information and Translational Sciences (HITS), 410 West 10th Street, Indianapolis, Indiana 46202 USA; Department of Medical and Molecular Genetics, Indiana University School of Medicine, Medical Research and Library Building, 975 West Walnut Street, Indianapolis, Indiana 46202 USA

## Abstract

Peste des petits ruminants (PPR), is an acute transboundary viral disease of economic importance, affecting goats and sheep. Mass vaccination programs around the world resulted in the decline of PPR outbreaks. Sungri 96 is a live attenuated vaccine, widely used in Northern India against PPR. This vaccine virus, isolated from goat works efficiently both in sheep and goat. Global gene expression changes under PPR vaccine virus infection are not yet well defined. Therefore, in this study we investigated the host-vaccine virus interactions by infecting the peripheral blood mononuclear cells isolated from goat with PPRV (Sungri 96 vaccine virus), to quantify the global changes in the transcriptomic signature by RNA-sequencing. Viral genome of Sungri 96 vaccine virus was assembled from the PPRV infected transcriptome confirming the infection and demonstrating the feasibility of building a complete non-host genome from the blood transcriptome. Comparison of infected transcriptome with control transcriptome revealed 985 differentially expressed genes. Functional analysis showed enrichment of immune regulatory pathways under PPRV infection. Key genes involved in immune system regulation, spliceosomal and apoptotic pathways were identified to be dysregulated. Network analysis revealed that the protein - protein interaction network among differentially expressed genes is significantly disrupted in infected state. Several genes encoding TFs that govern immune regulatory pathways were identified to co-regulate the differentially expressed genes. These data provide insights into the host - PPRV vaccine virus interactome for the first time. Our findings suggested dysregulation of immune regulatory pathways and genes encoding Transcription Factors (TFs) that govern these pathways in response to viral infection.

**Electronic supplementary material:**

The online version of this article (doi:10.1186/s13567-015-0153-8) contains supplementary material, which is available to authorized users.

## Introduction

Peste des petits ruminants (PPR), an acute viral disease of goats and sheep, characterized by fever, erosive stomatitis, conjunctivitis, gastroenteritis and pneumonia is caused by Peste des petits ruminants virus (PPRV). PPRV, a linear single stranded, negative sense RNA virus, belongs to the genus Morbilivirus, family Paramyxoviridae [1]. The PPRV genome of 15948 bp encodes six structural proteins, nucleocapsid (N), phosphoprotein (P), matrix (M), fusion (F), hemagglutinin (H) and large (L) protein in 3′ to 5′ direction (3′-N-P-M-F-H-L-5′); and two non-structural proteins (C/V) due to RNA editing of the phosphoprotein gene. Morbidity and mortality in this highly contagious disease is as high as 100% and 90%, respectively [1,2]. PPR was first reported in the Ivory Coast in 1942 and is now widespread across sub-Saharan Africa, Arabian Peninsula, Middle East and the Indian sub-continent. Given its economic relevance and severity, the disease is classified as a World Organisation for Animal Health (OIE) listed disease. Four different lineages of this virus (I–IV) have been defined all over the world based on molecular epidemiology of “N”, “F” and “H” gene sequences of the virus [3]. Viruses from Asia are predominantly classified into lineage IV and hence refereed as Asian lineage. However, few recent reports indicate that Asian lineage is now present in Africa as well [4].

In India, this transboundary contagious disease is endemic, causing severe economic losses to small ruminant production. PPR outbreaks have increased between the year 1996 and 2005 [5]. The economic loss was estimated to be around 1800 million Indian rupees (US$ 39 million) every year in India [6]. The number of outbreaks after 2005 has declined due to the mass vaccination efforts carried out from 2004 under the Assistance to States for Control of Animal Diseases programme, funded by the government of India. Three attenuated live cell culture vaccines, Sungri 96, Arasur 87 and Coimbatore 97 are commercially available in India. Sungri 96 (isolate of goat origin) was developed by Indian Veterinary Research Institute, Arasur 87 (isolate of sheep origin) and Coimbatore 97 (isolate of goat origin) were developed by Tamil Nadu Veterinary and Animal Sciences University. All these vaccines are considered to be potent and safe [7].

Sungri 96 vaccine, widely used in Northern India, was developed by attenuating the Sungri isolate of PPRV up to 60^th^ passage in Vero cells [5]. This virus, isolated from goat does not have a restriction in inducing immunity in either of the species, sheep or goat. Single dose of PPR live attenuated vaccine (Sungri 96) contains ~10^3^ TCID_50_ of Vero cell-attenuated PPRV and elicits robust immune response for up to 78 months [7], indicating that a single vaccination is sufficient to provide long-term immunity in sheep and goats. Cell-mediated immunity is suggested to play a major role in eliciting this response, which warrants further investigation. PPRV like other morbilliviruses is cell associated and reaches the other organs and the tissues by piggybacking on Peripheral blood mononuclear cells (PBMCs) [8]. PBMCs consist of immune cells T- and B-lymphocytes, as well as dendritic cells, which express SLAM, a (co) receptor for morbilliviruses. PBMCs are widely used as a model for studying the immunosuppressive nature, immune response and molecular pathways triggered following morbillivirus infection [9,10]. SLAM constitutively expressed on these cells facilitates the entry of Morbilliviruses. Basal level of SLAM expression in PBMCs has been shown to have high correlation with their ability to replicate PPRV [11]. Apart from SLAM, morbilliviruses use alternate receptors for entry. In addition to the SLAM, which acts as cellular receptor for PPR virus on immune cells, Ovine nectin-4 was identified as a novel epithelial receptor for PPR virus, which highlights on pathogenesis factors like tissue distribution and genetic variation of morbillivirus receptors [12]. CD46 (a membrane co-factor protein) acts as a cellular receptor for vaccine and laboratory passaged strains of measles virus. Understanding the molecular mechanisms of PPR vaccine virus (Sungri-96) induced protection, using PBMCs as a model will shed light on immune genes and cellular pathways involved in the protective mechanisms in hosts. This understanding of the host virus interactome would pave way for developing novel tools for better clinical management of PPR.

Recently, global transcriptome analysis was used to understand the molecular events of host vaccine interactome in Measles virus, Rinderpest virus [9,13]. However, no reports on PPRV-host interactome are available in the literature till date. Therefore, in the present study, as PPRV is lymphotropic we infected the peripheral blood mononuclear cells (PBMCs) isolated from goat with Sungri 96 vaccine virus and investigated the host-virus crosstalk vis-à-vis changes in global gene expression signatures at cellular level.

## Materials and methods

### Ethics statement and animals

The experimental procedures in the present study were approved by Institute Animal Ethics Committee (I.A.E.C No.F.1.53/2012-13-J.D.). Goat kids (5 months old) used for blood collection were housed in appropriate containment facilities with feed and water *adlibitum*.

### Virus and PBMC culture

PPRV strain-Sungri/96, a vaccine virus adapted to Vero cells (African Green Monkey Kidney cells), available at the Division of Biological Products, IVRI, Izatnagar and maintained at the passage level 60–62 was used in the present study. Blood was collected from goats (*n* = 5) in heparin coated vacutainer vials. The whole blood was layered on 3 mL of histopaque-1077 and was centrifuged at 2200 rpm for 40 min. The interphase layer rich in peripheral blood mononuclear cells (PBMCs) was transferred into a separate tube and was washed three times with RPMI-1640 medium at 1800 rpm for 10 min. The final pellet was re-suspended in a complete medium containing RPMI-1640 and 10% fetal calf serum (FCS). The live cells were counted in haemocytometer and 1 × 10^6^ cells per mL per well were seeded into two six well plates for culture in 5% CO_2_ incubator at 37 °C.

### PBMCs infection with PPRV and cytopathic effect

One plate served as a control (uninfected cells) and the other was used for infection. Sungri-96 vaccine virus, was grown in Vero cells and purified by banding on sucrose gradient (ultracentrifuged). The purified virus was titrated and tested for its infectivity in Vero cells and subsequently used in the experiment to infect PBMCs. Goat PBMC’s were infected with purified PPR virus (Sungri/96) at MOI of 1.0 in RPMI medium and incubated at room temperature for 1 h of adsorption. The cells were then washed, resuspended in complete RPMI medium and incubated at 37 °C in 5% CO_2_ incubator for 120 h. Cells were observed for morphological changes like clumping and ballooning at 24 h, 72 h and 120 h post infection (pi). As mRNA for N protein, is most abundantly transcribed and is required for viral replication and infection, at each of these time points, PCR and real-time PCR (qRT-PCR) for N gene expression was done to evaluate viral infection. Total RNA from cells was extracted using RNeasy mini Kit (Qiagen). The concentration and quality of the extracted RNA samples was determined using Bioanalyzer 2100 (Agilent Technologies Inc). The cDNA was synthesized using Revert Aid First Strand cDNA Kit (Thermo Scientific-#K1622) following manufacturers instructions. Fold change in qRT-PCR was calculated taking 24 h time point as calibrator. It was observed that the expression of N gene increased significantly from 24 h pi to 120 h pi. The viral infection in PBMCs at 120 h pi, was further confirmed by identifying PPRV specific proteins by western blot using polyclonal serum raised in goats against the Sungri/96 vaccine strain. Briefly, the cells after 120 h pi were harvested and lysed in lysis buffer (10 mM Tris/HCl, pH 8.0, 150 mM NaCl, 1% TritonX-100 and 1 mM DTT supplemented with protease and phosphatase inhibitors (2 mM sodium orthovanadate, 100 nM okadaic acid, 1 mM NaF, 1 mM β-glycerophosphate and cocktail (Sigma)). The protein samples were separated on 12% SDS-PAGE and electroblotted onto nitrocellulose membrane. After blocking overnight in 2% BSA the membrane was incubated with diluted polyclonal antiserum raised in goats (1: 100) for 1 h. The membrane was then washed three times with PBST buffer and then incubated in anti-goat IgG conjugated with HRP at a dilution of 1:10 000 for 1 h at 37 °C. The membrane was then washed thrice with PBST for 10 min and bands were visualized following incubation with diaminobenzidine tetrahydrochloride (DAB system, GeNei). PPRV specific bands confirmed viral infection in PBMCs after 120 h pi.

### Library preparation and RNA sequencing

Infected cells were harvested at 120 h pi along with the mock control cells. The harvested cells were pelleted at 2000 rpm for 10 min and stored in RNA later at −80 °C for RNA isolation*.* Total RNA was isolated from infected and mock-control cells using RNeasy Kit (Qiagen) according to the manufacturer’s instructions. RNA was quantified and checked for RNA integrity number (RIN) using Agilent 2100 Bioanalyser. As the RIN number for both the infected and control total RNA was ≥ 8.0, cDNA library was prepared using Ion Total RNA-Seq Kit according to the standard protocol and purified by magnetic bead module. The average fragment size of the library was observed to be 377 bp on the bioanalyzer. The beads were amplified by em-PCR after calculating the bead to fragment ratio. The amplified beads were enriched in Ion one Touch ES and the enriched template positive Ion sphere particles were subjected to sequencing using Ion 318 chip. The RNA sequencing data generated in FASTQ format was used for further analysis.

### Transcriptome quantification and functional analysis of differentially expressed genes

**Figure 1:**
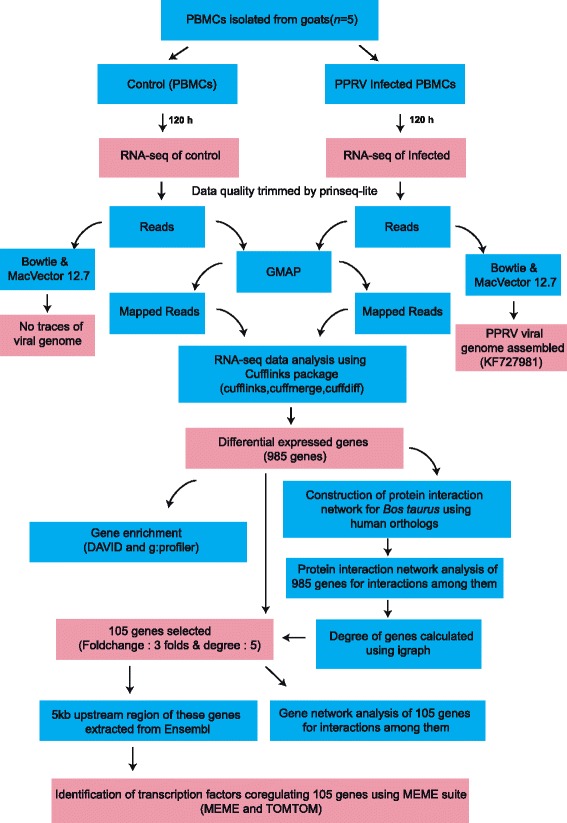
**Overview of the workflow.** PBMCs were collected from 5 goats and infected with PPRV virus at 1.0 mulitiplicity of infection (MOI). Uninfected PBMCs acted as control. RNA was isolated and sequenced from both infected and control PBMCs. The reads were quality filtered and mapped to the *Bos taurus* reference genome using GMAP program. Differential gene expression in infected vs control PBMCs was identified by subjecting the quality reads through the cufflinks package. Functional enrichment analysis was done using g:profiler and DAVID. Protein – protein interaction network was constructed for differentially expressed genes using the human reference interaction network and analyzed. Differentially expressed and highly connected (DHEC) 105 genes were selected and overrepresented conserved DNA motifs upstream of these genes were identified using MEME.TFs binding to these motifs were predicted using TOMTOM.

Figure 1 highlights the various steps followed in the present study. Ion torrent system-generated 160 bp raw single end reads were first processed by prinseq-lite.pl [14] to remove the reads of low quality (mean phred score < 25). Since annotated reference sequence of *Capra hircus* is unavailable, an integrated, non-redundant and well-annotated reference sequence of *Bos taurus* was used for further analysis. *Bos taurus* reference genome was earlier used for comparative analysis and functional annotation of goat transcriptome by several groups [15-17]. Also, the average sequence identity between these two species was estimated to be 82.79%. This identity increased to 93.77% if only exonic sequences were considered [17]. GMAP (Genome Mapping and Alignment Program) aligner was used to align all the quality reads to the *Bos taurus* reference genome downloaded from UCSC genome browser [18]. Gene expression was quantified using Fragments per Kilobase per Millions of reads (FPKM) values for each sample by utilizing the alignable reads in cufflinks [19]. The assembled transcripts for both the samples were merged using cuffmerge to get a merged transcriptome assembly, which provides a uniform basis for calculating gene expression. These merged transcripts were fed to cuffdiff to identify differentially expressed genes (Additional file 1) between uninfected PBMCs and PPRV infected PBMCs. Enrichment of Gene Ontology (GO) terms among differentially expressed genes was analyzed using g:Profiler [20]. Further, functional enrichment of these differentially expressed genes was performed using the Database for Annotation, Visualization and Integrated Discovery (DAVID, v6.7) [21]. All significant pathways (*P* ≤ 0.05) that were enriched in BioCarta, KEGG and Reactome databases were considered.

### Viral genome assembly

As PPRV is an RNA virus we could assemble the whole genome of PPRV (Sungri/96) from the infected PBMC transcriptome and not from the control transcriptome. The quality processed reads were aligned with the available PPRV reference genome from NCBI using bowtie 2.0 [22]. The aligned reads were assembled using MacVector 12.7.5 (@ 2012 MacVector Inc).

### Protein-Protein interaction network among the differentially expressed genes

The Biological General Repository for Interaction Datasets (BioGRID) is a curated biological database of protein-protein and genetic interactions created in 2003. It provides a comprehensive resource of protein–protein and genetic interactions for all major model organism species. BioGRID currently holds 347 966 interactions (170 162 genetic, 177 804 protein) curated from both high-throughput data sets and individual focused studies derived from over 23 000 publications in the primary literature [23]. In this repository, protein-protein interactions in human are well defined and those in *Bos taurus* are very few. Since protein interactions have been shown to be well-conserved across species [24], g:Orth in g:Profiler web server was employed to produce orthology (functionally equivalent genes) predictions between species to facilitate functional and interaction annotation transfer across species [20]. To construct the protein-protein interaction network with the differentially expressed genes in the present study, *Bos taurus* orthologs in human were queried using g:Orth. Customized perl scripts were used to extract interactions involving the differentially expressed genes. The complete interaction network visualized in Cytoscape 3.0.2 [25] was found to have an average degree of 4.4 with 6756 nodes and 30 023 edges (Additional file 2). Degree or connectivity of a node/gene in a network is the number of connections it has to other nodes/genes. The network involving only the 985 differentially expressed genes was found to comprise of 905 nodes and 5340 edges (Additional file 3). The network properties of each of these networks including clustering coefficient and graph density were calculated using igraph package in R [26]. Out of the 985 differentially expressed genes, 105 genes with 3 fold change in expression (positive or negative) and a degree of at least 5 were filtered to generate a sub-network using the original protein - protein interaction network. This set of 105 genes was designated as differentially expressed highly connected (DEHC) gene network.

### Extracting 5 kb upstream of DEHC genes

Ensembl is a comprehensive database addressing the challenges of decoding a eukaryotic genome from the set of functional elements it represents to providing access to the vast sea of data [27]. Ensemble BioMart is a hub for data retrieval across the taxonomic space. We extracted 5 kb upstream of 105 DEHC genes from Ensemble BioMart using the *Bos taurus* ensemble IDs. The sequences containing a string of undefined bases (N’s) due to partially complete contigs were eliminated from further analysis using a customized perl script.

### DNA motif discovery using MEME and prediction of transcription factors binding to upstream regions of DEHC genes

MEME (Multiple EM for Motif Elicitation) Suite is a comprehensive collection of tools most widely used for discovery of new transcription factor and protein domain binding sites [28]. The motif discovery algorithm MEME, finds ungapped motifs within DNA or protein sequences. TOMTOM [29] tool in the suite allows comparing the discovered motif to a database of motifs (JASPAR, JOLMA, etc.) to find matches to the established Position Weight Matrices (PWMs) of transcription factors. To predict TFs that bind to the upstream of the 105 DEHC genes identified in the present study, initially over-represented conserved motifs among these genes were identified using MEME followed by TOMTOM using a locally installed version of MEME suite. The TFs identified were mapped onto their orthologs in *Bos taurus*. The presence of these TF binding sites across the DEHC genes was depicted by a heatmap using cluster and Java tree view.

### Validation by Quantitative Real time PCR (qRT-PCR)

Quantitative Real time PCR was carried out on the same biological material that was used in RNA-Seq experiment. RNA was extracted from the harvested cells using RNeasy mini kit (Qiagen) and was quantified using nanodrop spectrophotometer (Thermo Scientific). cDNA was synthesized using Revert Aid First Strand cDNA synthesis kit according to the manufacturer’s instructions and qRT-PCR was performed using Applied Biosystems 7500 Fast system using 2X SYBR Green Master mix (USB, Sigma). Some DEHC genes were validated using GAPDH as an endogenous control. A panel of six housekeeping genes was tested for their stable expression across the infected and control samples at different time points in the experiment. RefFinder was used to generate stability values for all genes. GAPDH, was found to be the most stably expressed gene on analysis (Data not shown).

The primer sequences used in the study are given in Table 1. A melt curve analysis was performed to know the specificity of the qPCR. For the test and endogenous control genes the percentage efficiency ranged between 90% and 100%. All the samples were run in triplicates. The relative expression of each sample was calculated using the 2^−ΔΔCT^ method with control group as calibrator [30]. Student’s *t*-test was done in JMP9 (SAS Institute Inc, Cary, USA) and differences between groups were considered significant at *P* ≤ 0.05. Initially, Real time PCR validations were carried out on the same RNA for which RNA-Sequencing was done.

**Table 1:** **Genes and their primer sequence for validation**

**Genes**	**Primer sequence**	**Accession numbers**
RB1CC1	Forward: GAACCTCTCCACCAGCATGT	XM_005688971.1
	Reverse: GGTGAGGTAGCGGTTGTGAT	
YY1	Forward: GCAAGCCAAACTCTCCAGAC	XM_005695407.1
	Reverse: CCCAGACAGATCAGCAGTCA	
CD44	Forward: CCAGTCCCACACTGAAACCT	XM_005690117.1
	Reverse: GTCAGGCTTTGCTGAAGACC	
IFIT3	Forward: AAGGGTGGACACTGGTCAAG	XM_005698196.1
	Reverse: AGGGCCAGGAGAACTTTGAT	
VIM	Forward: CGCTCAAAGGGACTAACGAG	XM_005688054.1
	Reverse: TCCAGCAGCTTCCTGTAGGT	
CXCR4	Forward: GCCTGGTATCGTCATCCTGT	XM_005676186.1
	Reverse: TCGATGCTGATCCCAATGTA	

### Validation by qRT-PCR in goats vaccinated with Sungri/96 PPR vaccine

Animal experimentation in the present study was conducted under the approval mentioned above. Goats (*n* = 6, 5–6 months old), three served as control and three were used for vaccination with Sungri/96 vaccine virus. The goats were previously screened and were found to be negative for PPRV antibodies using c-ELISA. PBMCs were collected from both the groups (control and vaccinated) on 5^th^ day post-vaccination and differentially expressed genes validated in the in vitro study, were further evaluated for their expression in the in vivo experiment by qRT-PCR. The vaccinated animals were tested for presence of PPRV antibodies using c-ELISA and were found to be positive based on percentage inhibition (PI) values.

## Results

### PCR, Real time PCR, Western blot analysis and Viral genome assembly confirm viral infection in PBMCs

**Figure 2:**
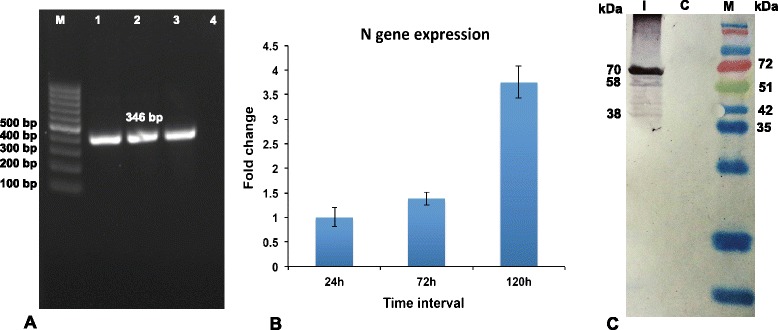
**Viral infection in goat PBMCs. A)** N gene amplification of 346 bp at 24 h (1), 72 h (2), 120 h pi (3), Negative control (4), M- 100 bp Ladder; **B)** Expression of N gene expression at 24 h, 72 h, 120 h pi by Real-Time PCR amplification. N gene expression at 24 h pi is taken as calibrator. Expression of N gene significantly (*P* ≤ 0.01) increased from 24 h pi to 120 pi indicating the increase in viral infection with time. **C)** Western blot of the infected (I) and control (C) cell lysate at 120 h pi with the polyclonal serum showed viral proteins of 38 kDa(M-protein), 58 kDa(N-protein), 70 kDa(HN-protein), Fusion (F) protein 60 kDa (not marked) between N and HN confirmed viral infection.

Viral infection in the PBMCs infected with PPRV (Sungri/96 vaccine virus) was confirmed by PCR and Real time PCR of N gene. An N gene amplicon of 346 bp could be amplified at all time points, 24 h, 72 h, 120 h post infection (pi) (Figure 2A). The N gene expression increased significantly from 24 h pi to 120 h pi (Figure 2B). PBMCs infected with PPRV also displayed robust cytopathic effects (rounding, blebbing, membrane fusion, clumping, ballooning and detachment) at 120 h pi. The viral infection in the present study was further confirmed by western blot of the infected cell lysate at 120 h pi, with the polyclonal serum (Figure 2C). The PPR virus (Sungri-96) whole genome was assembled from the infected transcriptome and the genome sequence is submitted to genBank NCBI with the accession no: KF727981 [31]. This ratified viral infection in PBMCs infected with PPRV.

### Functional analysis of differentially expressed genes reveals enrichment for immune regulatory pathways

**Figure 3:**
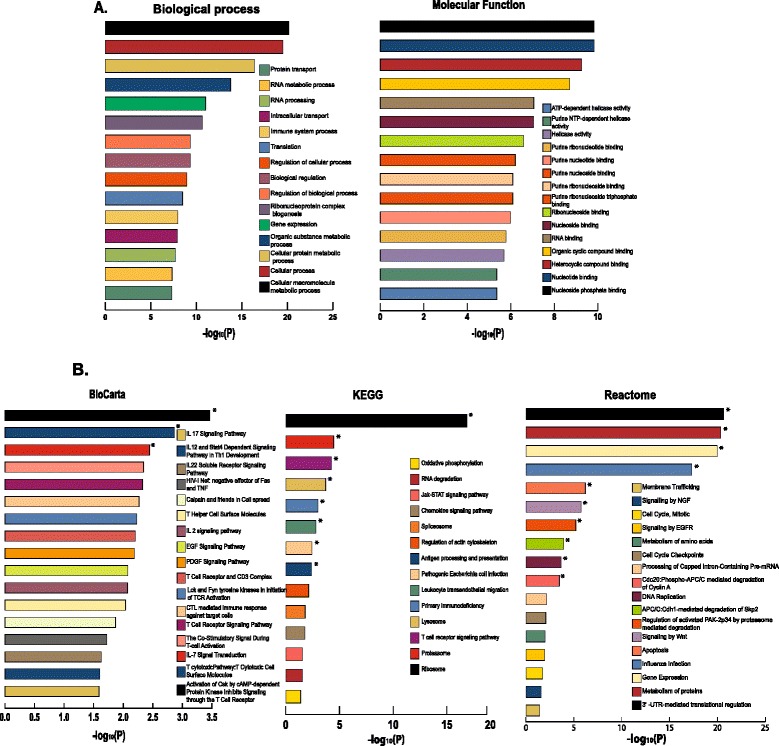
**Functional analysis of differentially expressed genes.** Functional annotation was carried out by using two bioinformatics tools, g:profiler and DAVID. **A)** Gene ontology (GO) terms were retrieved using g:profiler. The top 15 significantly (*P* ≤ 0.05) enriched GO terms in biological process and molecular function branches are shown. **B)** Enriched biological pathways were identified using DAVID. Significant (*p* < 0.05) categories among the canonical pathways found in KEGG, BioCarta, Reactome databases are shown. Categories with FDR < 5 are marked with *.

A total of 985 genes were differentially expressed in the infected PBMCs (Additional file 1). Annotation of functions of the differentially expressed genes was carried out by using bioinformatics tools, g:profiler [20] and DAVID [21]. Significant Gene Ontology (GO) terms for the differentially expressed genes were retrieved using g:profiler. The differentially expressed genes were distributed among more than 200 categories, belonging to the three branches of ontology namely biological process, molecular function, and cellular component (Additional file 4). Among biological processes, significant enrichment was found for the generic metabolic and cellular processes besides the immune system processes (Figure 3A). Nucleoside phosphate binding and nucleotide binding were observed to be enriched in the molecular function domain (Figure 3A). Among the 985 differentially expressed genes a total of 117 genes were found to be associated to the immune system processes exhibiting a significant enrichment (*p* < 1.11E-08) (Additional file 5).

The enriched biological pathways in infected PBMCs were identified using DAVID. Among the 985 differentially expressed genes (DEGs), DAVID provided functional annotation for 896 and 921 genes, associated to *Bos taurus* and *Homo sapiens*, respectively. A total of 268 genes were mapped to 14 statistically significant categories (*p* < 0.05; Figure 3B, Additional file 6) among the documented canonical pathways found in KEGG. Ribosome, proteasome and T-cell signaling pathways were the top three annotated with 36, 14 and 22 genes, respectively. There were also a statistically significant number of mapped genes representing antigen processing and presentation, spliceosome, chemokine and JaK-STAT signaling pathways. When mapped to the signaling pathways in BioCarta, 128 differentially expressed genes representing 18 statistically significant categories with most of them related to immune system regulation were detected (*p* < 0.05; Figure 3B, Additional file 6). Out of the 985 differentially expressed, a total of 576 genes could also be mapped to 19 significant (*p* < 0.05) categories in the Reactome database. Among these categories, gene expression, metabolism of proteins and 3′-UTR-mediated translational regulation were found to be over-represented with 90, 69 and 48 genes, respectively (Figure 3B, Additional file 6).

### Dense cross-talk in the protein - protein interaction network of differentially expressed genes is disrupted in infected state

The global interaction network of the 985 differentially expressed genes (GN) and the network involving interactions only between the 985 differentially expressed genes (DEN) were both constructed and analyzed (see Materials and methods). Both the graph density and the average clustering coefficient for the DEN was found to be much higher than that observed for GN (Graph Density: 0.013 vs 0.001; and Clustering Coefficient: 0.066 vs 0.026). Among the 985 differentially expressed genes 407 genes were upregulated and 578 genes were downregulated. The average connectivity was found to be 15.43 and 9.25 for the up- and downregulated genes, respectively. Though, the degree distribution was significantly different (*p* = 2.983e-05; Wilcox test), functional enrichment of the up and downregulated genes, independently, revealed significant enrichment of similar cellular, metabolic and immune regulatory processes.

**Figure 4:**
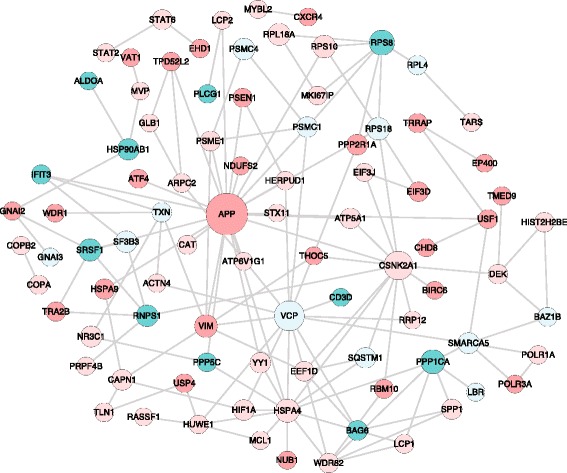
**Protein–protein interaction network for differentially expressed highly connected genes (DEHCNet).** Genes that were upregulated and downregulated were shown in green and red colors, respectively, with the gradient showing the extent of expression (log_2_(fold change) ranging from 1.5 to 5.15 for upregulated and −1.5 to −3.39 for downregulated genes). The diameter of the node represents the connectivity/degree of the node among the 985 differentially expressed genes. Self loops have been removed. CARD11, IRF3, RAC1, UBA2, CLINT1, HERC2, TMEM66, DCAF8, BTG1, MAT2A, HYOU1, POLA1, CD44, ID2 and RB1CC1 genes had no connectivity with other DEHC genes.

Using the 985 differentially expressed genes and the corresponding protein-protein interaction network between them, a total of 105 genes with 3 fold change in expression (positive or negative) with a degree of 5 were filtered to finally represent the protein - protein interaction network. These genes were designated as Differentially Expressed Highly Connected (DEHC) genes. This resulted in a set of 237 interactions in this DEHCNet as illustrated in Figure 4. Genes, RPS8, PP1CA, BAG6, PPP5C and VCP were found to be upregulated and APP, EP400, NUB1, MK1671P, TPD52L2, VIM, IFIT3, CD3D and GNAI3 were found to be downregulated (Table 2).

**Table 2:** **Genes in the DEHCnet involved in cell profileration, apoptosis and immune regulatory pathways**

**Gene**	**Function**	**Reference**
CSNK2A1	Plays key role in cellular growth and differentation. Suppressor of Apoptosis	[33]
EP400	Promotes cell cycle progression and inhibits induction of apoptosis or senescence.	[34]
NUB1	Role in cell cycle progression.	[35]
MKI67IP	Interacts with Ki-67 in proliferating cells and has a role in mitosis	[36]
TPD52L2	Regulator of cell proliferation. Marker for breast cancer and acute lymphoblastic leukemia.	[37]
RPS8	Role in apoptosis. Reacts with CDK11p46 and sensitizes cells for Fas ligand-induced apoptosis	[38]
PPP1CA	Regulates cell cycle progression and apoptosis.	[39]
BAG6	Represses function of Tim3 expressed on T cells during Hepatitis C virus infection. Regulates Apoptosis.	[40]
PPP5C	Regulates signaling cascades that suppress growth or induces apoptosis.	[41]
IFIT3	Induces antiviral response (anti-viral signaling) by inducing IFN-alpha.	[42]
CD3D	Involved in T cell development. Mutation in this gene leads to Immunodeficiency (SCID)	[43]
APP	Signaling molecule Involved in synaptic adhesion. Processed to form ß-amyloid peptides	[44]
VCP	Chaperon protein that regulates DNA damage and repair	[45]

In addition we found several major classes of regulators at transcriptional, signaling and post-transcriptional levels dysregulated in the DEHC network. For instance, 3 transcriptional factors viz. CAT, IRF3 and YY1 as well as important signaling proteins, TRRAP – a phosphoinositide 3-kinase, CARD11- a membrane-associated guanylate kinase, PRPF4B – a CDK like kinase and CSNK2A1- casein kinase II were found to be significantly altered. Also, our DEHCNet analysis uncovered 16 RNA binding proteins (RBPs) viz. RPS8, RPS10, RPS18, SRSF1, RPL18A, RNPS1, DEK, EIF3D, RPL4, TRA2B, MKI67IP, RBM10, HUWE1, LBR, PRPF4B and RRP12 that are involved in post transcriptional regulation of gene expression [32].

### Dysregulated TFs contribute to rewiring the expression landscape of the infected PBMCs

**Figure 5:**
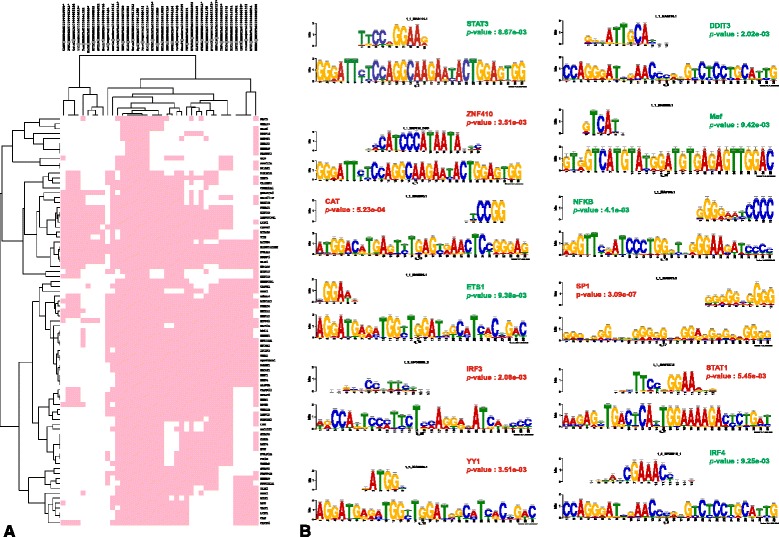
**Transcription factors binding upstream of DEHC genes. A)** Heatmap showing the binding sites of 41 *Bos taurus* orthologs upstream of DEHC genes. **B)** Motif alignments of each of the predicted 12 motifs with their corresponding known binding motifs for TFs, which were also found to be differentially expressed. The bottom sequences are the predicted motifs and the top sequences are TF-binding consensus sequences matching the motifs. Binding specificity of TFs is shown for all the motifs. Genes encoding the TFs that are upregulated are shown in green colour and that are downregulated are shown in red colour.

As transcription factors (TFs) are the key regulators of gene expression that bind directly to the upstream regions of genes, we identified the transcription factors among the 985 differentially expressed genes that contribute to the transcriptional control of the 105 DEHC genes. Overrepresented conserved motifs among the 105 DEHC genes were identified using MEME (Multiple EM for Motif Elicitation) [28] and TFs binding to these overrepresented motifs were predicted using TOMTOM [29] (see Materials and methods). A total of thirty overrepresented motifs were predicted (Additional file 7), out of which the top ten significant motifs are shown in Table 3. A total of 198 transcription factor Position Weight Matrices (PWMs) were predicted to bind to the thirty overrepresented motifs identified in the upstream regions of 83 genes. Among these 198 only 41 TFs were found to have orthologs in *Bos Taurus* genome (Figure 5A). Among these 41 TFs, 12 TFs (CAT, DDIT3, ETS1, IRF3, IRF4, MAF, NFKB1, SP1, STAT1, STAT3, ZNF410, YY1) were a subset of the 985 differentially expressed genes (Figure 5B). Genes, CAT, IRF3, PRDM1, SP1, STAT1, ZNF410 and YY1 were downregulated and DDIT3, ETS1, IRF4, MAF, MSN, NFKB1 and STAT3 were upregulated (Figure 5B). Out of these 12 TFs, CAT, IRF3 and YY1 were found in the DEHCNet with a connectivity of 13, 10 and 21, respectively (Additional file 8).

**Table 3:** **Motifs upstream of 105 DEHC genes and predicted TFs that bind to these motifs**

**Sl.No**	**MEME Motif**	**Significance**	**Sites**	**Transcription factors**
1	GGGATTCTCCAGGCAAGAATACTGGAGTGG	6.8e-576	59	NFKB1, RELA, MOT3, NFATC2, dl_2, dl_1, REL, Stat3, E2F7_DBD*, NKX2-8_DBD*, NFATC1_full_3*, NKX2-8_full*, ZNF306_full*, ZNF410_DBD*, DPRX_DBD_2*
2	ACCCCATGGACTGCAGCCTACCAGGCTCCT	1.2e-472	59	EBF1, REST, ADR1, YPR022C, REST, Smad3_secondary, RUNX2_DBD_2*, RUNX3_DBD_3*, Vdr_DBD*, EBF1_full*, VDR_full*
5	TGGGGTCGCAAAGAGTCGGACACGACTGAG	1.4e-424	56	Hoxc12_3480.1, ADR1, usp, Hoxc10_2779.2, Sp4_secondary, Rfx3_secondary, FOXK1_DBD*, RARG_DBD_3*, HOXC12_DBD_2*, HOXC11_full*, HOXD12_DBD_2*
4	ATGGACAGAGGAGCCTGGTGGGCTGCAGTC	1.6e-419	57	Zfp691_secondary, NRG1,ESR1, Smad3_secondary, Pax5, Zbtb7b_secondary, Myf6_secondary, Zic3_secondary, TFAP2A_DBD_3*, TFAP2C_full_2*, KLF14_DBD*, KLF13_full*, Tcfap2a_DBD_3*, SP8_DBD*
3	TAAGTCGCTCAGTCGTGTCCGACTCTTTGC	2.2e-411	60	Sp4_secondary, Egr1_secondary, FOXK1_DBD*, PAX5_DBD*, PAX2_DBD*, PAX1_DBD*, Foxk1_DBD*
6	AGGAAATGGCAACCCACTCCAGTATTCTTG	3.6e-380	44	RFX1, Hic1_primary, Duxl_1286.2, FEV, Titf1_1722.2, IRF2, Irf3_primary, Gamyb, Nkx2-4_3074.1, PPARG, IRF3_full*, Hic1_DBD*, Hic1_DBD_2*, NKX2-8_DBD*, NKX2-8_full*
7	TTGCCATTTCCTTCTCCAGGGGATCTTCCT	5.5e-357	55	ELF5, Rfxdc2_secondary, NFATC2, REI1, FEV, YRM1, SPI1, STAT1, EBF1, Elf3_primary, IRF3_full*, FOXB1_DBD*, ETV6_full*
8	CCAGGGATCGAACCCAGGTCTCCTGCATTG	6.60E-282	57	Zfp187_secondary, Ddit3::Cebpa, SOK2, EBF1, Hnf4a_primary, Zic1_secondary, PUT3,Zic2_secondary, Esrra_secondary, Irf4_primary, ZNF524_full_2*, ZIC4_DBD*, ZIC1_full*, RORA_DBD*, Zic3_DBD*
9	TTCTTAACCACTGAGCCACCAGGGAAGCCC	1.70E-261	54	NF-kappaB, EBF1, SPI1, PUT3, GCR2, Bapx1_2343.1, Nkx3-1_primary, Lag1, GCR1, REL, EBF1_full*, Tcfap2a_DBD_3*, ZBTB7B_full*, MAFK_DBD_2*
10	GGGTTCGATCCCTGGGTTGGGAAGATCCCC	5.50E-254	45	SPI1, dl_1, Ddit3::Cebpa, RELA, EBF1, NF-kappaB,dl_2,REL, NFKB1,PUT3, FOXO3_full_3*, DPRX_DBD_2*, RHOXF1_DBD*, RHOXF1_full*, RHOXF1_full_2*

*Predicted transcription factors using Jolma database other than those predicted using JASPER database.

### Validation of RNA sequencing data by real-time RT-PCR

**Figure 6:**
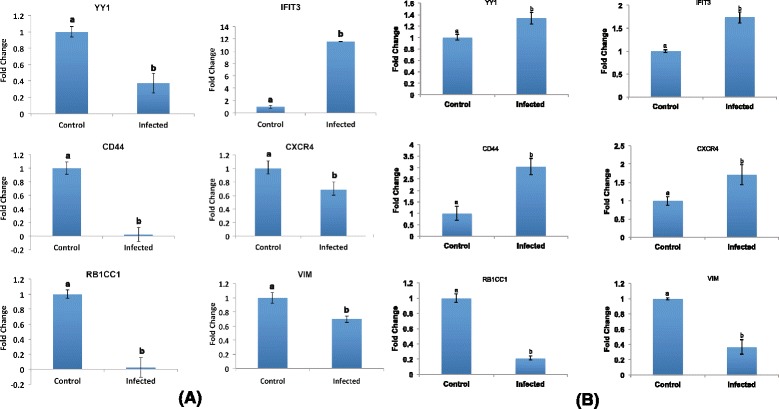
**mRNA levels of genes validated using quantitative real-time PCR. A)** In vitro experiment – PBMCs infected with Sungri/96. **B)** In vivo experiment – Goats vaccinated with Sungri/96. Fold change (2^−ΔΔCT^) with control as the calibrator is represented along with the standard error of difference. Levels not connected by same letter are significantly (*P* ≤ 0.05) different.

Six differentially expressed genes having important roles in immune regulation and involved in viral entry were validated with real time RT-PCR. These included transcription factor YY1, interferon induced anti-viral protein IFIT3, cell surface glycoprotein CD44, chemokine receptor CXCR4, intermediate filament VIM, RB1CC1 an apoptosis and autophagy regulator. All the six differentially expressed genes (YY1, IFIT3, CD44, CXCR4, RB1CC1 and VIM) validated were in concordance with RNA sequencing results (Figure 6A). The real-time results revealed same pattern of transcription as RNA sequencing data (Table 4). These differentially expressed genes were further evaluated for their expression in vivo in vaccinated goats (see Materials and methods). IFIT3, VIM and RB1CC1 showed same pattern of expression as in vitro whereas CD44, downregulated in vitro was found to be upregulated in vivo, CXCR4 and YY1, which were found to be downregulated in vitro, were upregulated in vaccinated goats (Figure 6B).

**Table 4:** **Validation of RNA sequencing data and fold change comparison**

**Genes**	**RNA Sequencing fold change**	**Real-time RT-PCR fold change**
IFIT3	+5.336	+11.47
RB1CC1	- 10.49	- 43.11
CD44	−4.49	- 44.94
CXCR4	−8.99	- 1.43
VIM	−4.99	- 1.44
YY1	−2.99	- 2.73

“+” Indicates up-regulated gene and “-” Indicates down-regulated genes.

## Discussion

In this study our approach to dissect the host-virus interactome broadly comprised of the following steps as shown in the flowchart (Figure 1) and discussed in Materials and methods: 1) Confirming the viral infection 2) RNA-sequencing for comparative transcriptomic analysis in infected and uninfected goat PBMCs 3) Functional analysis to identify the processes enriched in differentially expressed genes and uncovering the associated protein interaction network and 4) Discovery of the transcription factors contributing to the dysregulation of genes.

Viral infection in the PBMCs infected with PPRV at all time points (24 h, 72 h and 120 h pi) was confirmed by amplification and expression of N gene, which is most abundantly transcribed and required for PPR viral replication and infection [46]. The cytopathic changes in the PBMCs infected with PPRV corroborated the view that PBMCs were infected by the virus. Similar cytopathic effect was reported from 4^th^ day pi, in Sungri vaccine virus strain infected Vero cells [47]. PPRV infection in PBMCs was further confirmed by western blot analysis of PPRV specific proteins using antibody raised against purified virus as documented earlier [48]. The assembly of the PPR viral genome from the RNA-seq reads of the infected PBMCs ratified the viral infection. Our ability to build the complete viral genome based on the transcriptome of the infected PBMCs strengthens the notion that the transcriptomes of blood as well as other body fluids could be used as surrogates to know the extent of infection by various pathogens. To our knowledge this is the first global analysis demonstrating the feasibility of building a complete non-host genome from a blood transcriptome.

Analysis of transcriptome data generated from 120 h pi of PBMCs infected with PPRV revealed 985 differentially expressed genes. On assigning the gene ontology terms using g- profiler 117 genes exhibited a significant enrichment to the immune system processes. The enrichment of T-cell signaling [49], chemokine [50], antigen processing and presentation [51], Jak-STAT [52], IL-7 [53] pathways on annotation by DAVID, indicated the involvement of immune response regulatory pathways in PBMCs infected with PPRV. In this study, we found that PPRV infection induced, interferon regulatory factors - IRF4 and IRF5, interferon induced tetricopeptide - IFIT3 (ISG60) and tripartite motif protein - TRIM56. It has been reported that Interferon regulatory factors and TRIM56 are activated in response to invading viruses (ssRNA or dsRNA viruses) and lead to increase in transcription of interferon stimulated genes (ISGs) by binding to the interferon stimulated responsive elements (ISREs). TRIM56, a virus inducible E3 ubiquitin ligase, was found to restrict bovine viral diarrhea virus replication by inducing ISGs that are transcriptionally regulated by IRFs [54]. Thus, TRIM proteins together with IRFs play an important role in innate immunity to virus infections by stimulating interferon stimulated genes [55]. This suggests that TRIM56, IRF4/5 may act in restricting PPRV replication by inducing ISGs (IFIT3/ISG60) thus augmenting the innate immune response. However, to identify the role of transcriptome signatures in understanding cross protection with different PPRV strains, independent studies have to be conducted for each of the strains under consideration to identify common signatures across different strains. These common signatures may then help in understanding cross protection within different PPRV strains as studied in other viral strains [56].

Protein interaction network (interactome) analysis provides an effective way to understand the interrelationships between genes [57]. The graph density and the average clustering coefficient for the network involving interactions only between the 985 differentially expressed genes (DEN) was found to be much higher than that observed for the global interaction network of the 985 differentially expressed genes (GN) suggesting that the network of interactions between differently expressed genes is much more intricately connected and is likely contributing to the observed phenotype due to the disruption in the interactome. High clustering coefficient of the DEN compared to the GN also suggests that the former network is more modular. The average connectivity among the 407 genes that were upregulated and 578 genes that were downregulated among the differentially expressed genes was significantly different. However, similar functional enrichment of the up and downregulated genes, independently, suggested no evidence of functional differences in the gene pool contributing to these differences. Nevertheless, it is possible to speculate from this data that up-regulated genes due to their already high number of interactions might be increasing their interactome while down-regulated genes might be losing their functionality thereby contributing to the overall disruption of the protein-protein interactions in the infected state.

The DEHC network represented genes with a fold change ≥ 3 and degree ≥ 5 among the differentially expressed genes. Genes involved in cell proliferation such as EP400, NUB1, MK1671P and TPD52L2 were found to be downregulated while genes such as RPS8, PP1CA, BAG6 and PPP5C involved in progression of apoptosis were upregulated (Table 2). VIM, Viementin-involved in maintaining cell shape and integrity was also found to be downregulated. These findings were in line with the ability of PPRV virus to induce apoptosis in goat PBMCs [58]. IFIT3 that induces IFN-γ antiviral response, CD3D that is involved in T-cell development and GNAI3, critical for normal B cell function were all upregulated reasserting the involvement of the immune response regulatory pathways in PPRV infected PBMCs.

APP, which is a precursor of β-amyloid protein - known for its pro-inflammatory activity, was downregulated. Our analysis identified 147 and 25 interacting proteins for APP among the 985 differentially expressed genes and 105 DEHC genes, respectively (Additional file 8). This observation supports the notion that some viruses suppress the expression of proinflammatory cytokines thereby potentially impeding the antiviral host response to infection [59]. Chaperone protein, VCP that regulates DNA damage and repair was also upregulated. It was found to interact with 16 proteins in the DEHCNet and 77 proteins among the 985 differentially expressed genes. It was recently established that VCP is a host factor required for viral RNA replication of polio virus and that it provides a novel link between cellular protein secretion and viral RNA replication [60] suggesting its probable role in PPRV infected PBMCs. Further, the presence of 16 dysregulated RNA Binding Proteins (RBPs) reaffirmed the enrichment of spliceosome pathway among the KEGG pathways.

Transcription factors (TFs) are key regulators of gene expression and 12 TFs were identified among the 985 differentially expressed genes. TFs, IRF3 [61], IRF4 [62], MAF [63], NFKB1 [64], PRDM1 [65], SP1 [66], STAT1 [67] and YY1 are involved in the immune regulatory pathways strongly supporting the observation that immune system associated genes are differentially expressed in PPRV infected PBMCs.

An analysis of the overlap of the 41 TFs identified from footprinting the upstream regions above, among the 985 differentially expressed genes, indicated a strong enrichment for TFs in this set (*p* = 1.34E-05, Hypergeometric test). As the activity of TFs is governed by a number of factors including their protein levels, sub-cellular localization and post-translational modifications, the RNA levels may not always explain their activity. Our analysis and the observations presented here nevertheless provide a strong support that the 12 identified TFs could be significantly contributing to rewiring the host transcriptomic landscape under PPRV infection. Some of the 41 identified TFs likely controlling the expression of the differentially expressed genes may not always be differentially expressed because of our thresholds and/or they are farther upstream in the complex hierarchical transcriptional network [68] and hence their effect may not be as direct.

The quantity of data generated from RNA sequencing is large and therefore it is important to validate differential expression by real-time RT-PCR. Real time results in our study were found to be consistent with RNA sequencing analysis, but, we observed fold variations between two methods, which could be due to intrinsic differences between two techniques. Further, expression of in vitro validated genes (IFIT3, RB1CC1, VIM, CD44, CXCR4 and YY1) when evaluated for their expression in vaccinated goats (in vivo) showed similar expression pattern for IFIT3, RB1CC1 and VIM. However, CD44, CXCR4 and YY1 all of which were downregulated in in vitro were found be upregulated in vivo. This shows there could be a considerable difference in expression pattern of differentially expressed genes seen in vitro from in vivo experiments. Thus, it is necessary to analyze the expression of molecules which are predicted as anti-viral or pro-viral in, in vitro studies for their similar functions in vivo as in in vivo experiments the regulation of these molecules might be different due to a multitude of processes and interactions.

In this study, we investigated the host-virus interactome underlying PPRV vaccine virus infection. RNA-seq data analysis followed by functional analysis revealed enrichment of immune system regulatory pathways in PBMCs infected with PPRV. IFIT3 and TRIM56 can be suggested as the important early innate immune molecules following PPRV infection. The protein-protein interaction network of differentially expressed and highly connected genes revealed key dysregulated genes involved in immune system regulatory pathways, spliceosome pathways and apoptotic pathways. Genes encoding TFs that co-regulate the differentially expressed and highly connected genes were identified among the differentially expressed genes. Most of these TFs were found to govern immune regulatory pathways. Our results show that one can build the complete viral genome based on the transcriptome of the infected PBMCs indicating that the transcriptomes of blood as well as other body fluids could be used as markers to the know the extent of infection by various pathogens. This observation extends on recent studies on the impact of non-host genomic signals in the transcriptomes of humans and other hosts in uncovering the host-pathogen interplay [69].

## Additional files

Additional file 1:
**List of differentially expressed genes and their fold change.** This additional file shows list of 985 differentially expressed genes and the fold changes associated with them. Additional file 2:
**Global interactions of 985 differentially expressed genes.** This additional file shows global interaction of 985 genes with 6756 nodes and 30023 edges. The nodes and the edges were identified using cytoscape 3.0.2. Additional file 3:
**Interactions of 985 differentially expressed genes among themselves.** This additional file shows protein interactions between 985 differentially expressed genes with 905 nodes and 5340 edges. The nodes and the edges were identified using cytoscape 3.0.2. Additional file 4:
**GO terms retrieved from g-profiler for 985 differentially expressed genes.** This additional file shows the significantly enriched processes in three GO terms namely biological process, molecular function and cellular component. The genes involved in each process and their significant *p*-value has been indicated in this file. The GO terms showed significant enrichment of immune system process in addition to the other cellular processes. Additional file 5:
**Top 15 enriched processes in biological process and molecular function ontology for 985 differentially expressed genes.** This additional file shows top 15 significantly enriched processes in biological process and molecular function. Additional file 6:
**Significant terms mapped to KEGG, BioCarta and Reactome databases.** The additional file 4 shows canonical biological pathways enriched in KEGG, BioCarta and Reactome. Pathways mainly involved in regulating immune system process were enriched with significance *P* < 0.05. Additional file 7:
**Predicted TFs that bind to upstream regions of DEHC genes.** This additional file shows 30 overrepresented conserved motifs among 105 genes identified by MEME suite and the predicted transcription factors (TFs), which binds to these motifs using TOMTOM. Additional file 8:
**Connectivity or degree of 105 genes among the 985 differentially expressed genes and DEHC network.** This additional file gives the degree of genes among the 985 differentially expressed genes and degree of genes among the 105 differentially expressed highly connected network.

## Abbreviations

PPR: Peste des petits ruminants
PPRV: Peste des petits ruminants virus
PBMCs: Peripheral blood mononuclear cells
TCID: Tissue culture infective dose
GMAP: Genomic mapping and alignment program
FPKM: Fragments per Kilobase per Millions of reads
DAVID: Database for s
DEHC: Differentially expressed and highly connected genes
TFs: Transcription factors
MEME: Multiple em and motif elicitation
PWMs: Position weight matrices
